# A case report of an unusual presentation of chyle leak after repair of a perforated gastric ulcer

**DOI:** 10.1016/j.ijscr.2025.112084

**Published:** 2025-10-20

**Authors:** Muhammed Isa Bin Jusoff Albar, Han Boon Oh

**Affiliations:** a Department of General Surgery, Sligo University Hospital, Ireland; b Department of General Surgery, Ng Teng Fong General Hospital, Singapore; c Department of General Surgery – Division of Breast and Endocrine Surgery, Ng Teng Fong General Hospital, Singapore

**Keywords:** Chyle leak, Perforated gastric ulcer, Omental patch repair, Case report

## Abstract

**Introduction and importance:**

Chyle leak is an uncommon complication of abdominal surgeries. There are no reported cases of a chyle leakage following an omental patch repair for a perforated gastric ulcer before. A chyle leak from such a case however can successfully be treated conservatively.

**Case presentation:**

We report an unusual case of a 57-year-old male presented with a perforated gastric ulcer who underwent a laparotomy and omental patch repair, which subsequently presented with a chyle leak from his abdominal wound.

**Clinical discussion:**

Post operative chyle leak usually occurs from the intraoperative disruption of the thoracic duct, cisterna chyli, or major tributaries of the lymphatic system, and hence can also occur following abdominal and retroperitoneal surgeries. Chyle leakage is not one of the known postoperative complications following an omental patch repair. As chyle leak after omental patch repair has not been reported before, we sought to ascertain the causes. This case was successfully treated conservatively. This patient had a low volume chyle leak. The main stay of conservative treatment for a low volume chyle leak is to reduce chyle production via nutrition optimisation. Literature found that a more conservative approach to management of a chyle leak can be very successful. We discuss the conservative management of the chyle leak with intraabdominal drainage and a very low-fat diet.

**Conclusion:**

A chyle leak following an omental patch repair for a perforated gastric ulcer is an unusual post-operative complication. It can be treated conservatively successfully provided the chyle leak maintains at a low volume.

## Highlights

• We report a first case report of a chyle leakage following an omental patch repair for a perforated gastric ulcer.
• We discuss the management of the chyle leak with intraabdominal drainage and a very low-fat diet.
• It can be treated conservatively successfully provided the chyle leak maintains at a low volume.

## 1 Introduction

A perforated gastric ulcer is one of the most common causes of an acute abdomen presentation to the emergency department, and is a surgical emergency. The surgical management of a perforated gastric ulcer is commonly through stomach-preserving surgery including omental implantation or simple closure [13]. The incidence of a chyle leak following an intraabdominal surgery range between 0.29 and 16.3 % depending on the type of surgery performed, with the highest incidence rate following a pancreatic resection [9]. However, there are no recorded reports of a chyle leak following an omental patch repair for a perforated gastric ulcer. We present a first case of a perforated gastric ulcer which underwent a laparotomy and omental patch repair, which subsequently re-presented back with a chyle leak. This case report has been reported in line with the SCARE checklist [5].

## 2 Presentation of case

**Fig. 1:**
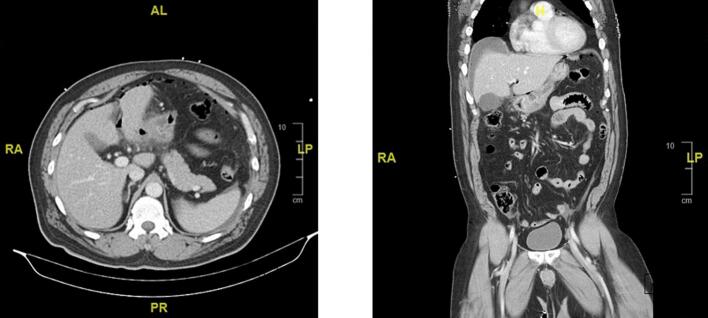
Computed Tomography showing diffuse wall thickening of the distal stomach, with associated perigastric fat standing, free fluid, and pneumoperitoneum.

A 57-year-old Chinese man presented to the emergency department with a 1-day history of severe abdominal pain and shortness of breath. Two days prior to presentation, he was involved in a road traffic accident where he was a motorbike rider colliding with a lorry, of which he claimed there was no direct injury to the abdomen. Physical examination at the time demonstrated a tender upper abdomen with localised guarding. Patient's past medical history included gout, and he was obese with a BMI of 32.9 kg/m^2^. There was no significant family history to note. Patient was a non-smoker, and no reported alcohol, or recreational drug use. In the emergency department he was hypertensive with a blood pressure of 186/100 mmHg and tachycardic with a heart range ranging between 90 and 110 beats per minute. Initial blood investigations noted a significant haemoglobin level of 8.8 g/dL, with a total white blood count of 22.67 × 10^9^/L, and a raised serum lactate of 3.0 mmol/L. A stat contrast-enhanced CT abdomen and pelvis was done which showed diffuse wall thickening of the distal stomach with a possible rent in the anterior wall of gastric pylorus associated with perigastric fat standing, free fluid and pneumoperitoneum (Fig. 1); all of which were suspicious of a perforated gastric ulcer.

We proceeded with an emergency exploratory laparotomy which revealed a benign looking 1x1cm perforated anterior pre-pyloric ulcer. Only minimal contamination with gastric juice was noted with no chyle seen during the surgery. A thorough intraperitoneal washout was done, and intraoperatively a tongue of healthy viable omentum was brought up as a patch and was secured with 5 interrupted 3/0 PDS sutures as per a Cellan-Jones repair [6]. Post-operatively patient developed a hospital-acquired pneumonia which was treated with antibiotics. Patient was well and discharged for home on post-op day 7 after clearance from physiotherapy and occupational therapy, and was scheduled for an early follow up in the outpatient clinic.

During the surgical clinic follow up review on post-op day 19, patient complained of pus-like discharge over the abdominal wound with wound dehiscence, and abdominal pain. Physical examination at this time noted wound dehiscence with what seemed like pus discharge that communicated with a deeper intraperitoneal cavity. He was otherwise clinically well, afebrile and did not show any signs of sepsis. Patient was admitted from clinic and blood investigations revealed a total white blood cell count of 11.96 × 10^9^/L with a C-reactive protein of 56.9 mg/L.

We then proceeded with a wound exploration under anaesthesia which showed wound and fascia dehiscence of the laparotomy wound, with copious amount of chyle-like fluid in a sealed subhepatic collection. The initial omental patch repair was intact. No bile or gastric content was noted during this surgery. Thorough intraperitoneal washout, and a vacuum-assisted closure dressing was applied with the intention of reexploring later. On post-op day 2, a relook surgery revealed minimal fluid in the cavity. A washout was carried out, and two 19F Blake drains were inserted in the left hypochondrium cavity, and the other into the subcutaneous region of the wound. The abdomen was then closed.

**Fig. 2:**
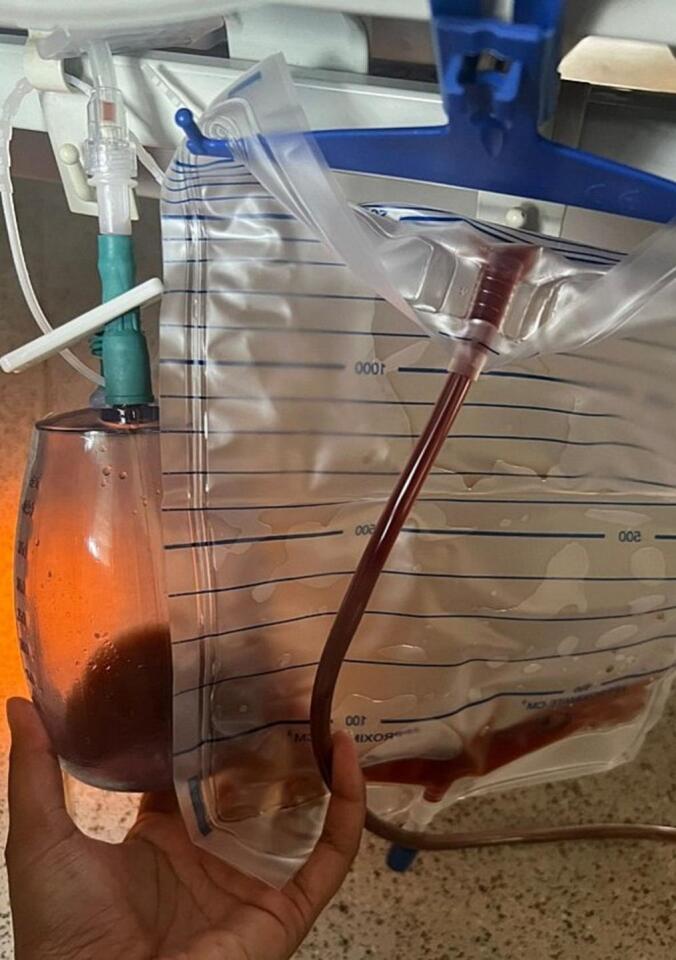
Hemoserous fluid drained from intraabdominal and subcutaneous drains on post-op day 2.

**Fig. 3:**
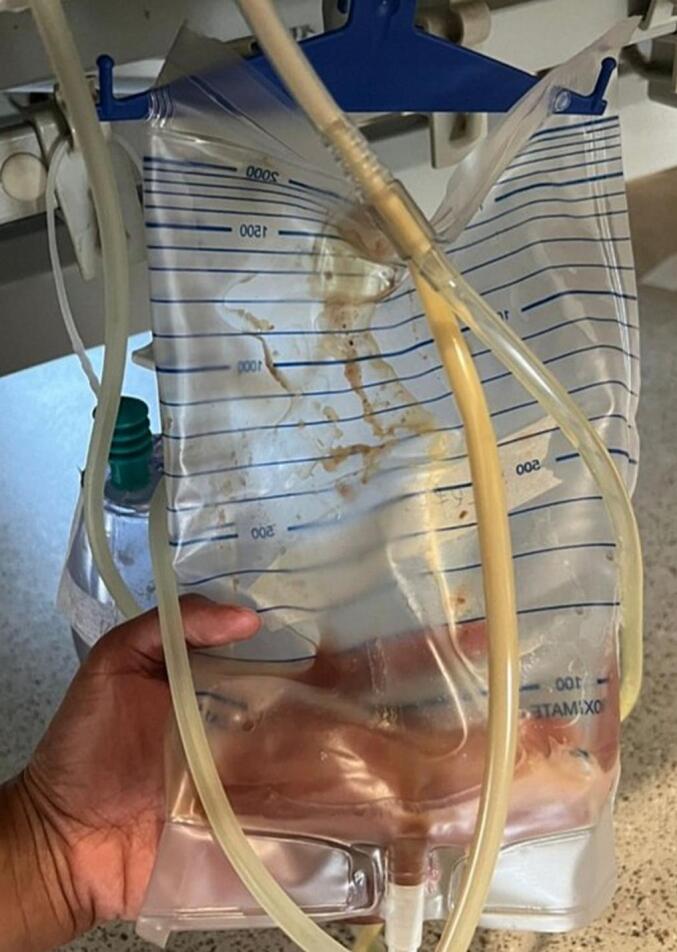
Milky turbid fluid drained from intraabdominal and subcutaneous drains on post-op day 3 after diet was re-established.

Post-operatively, patient was recovering well with both drains draining hemoserous fluid (Fig. 2). Patient was allowed full feeds on post-op day 2, prior further escalating to soft diet on post-op day 3. Once feeding was established, it was noted that the drains started draining chylous fluid again (Fig. 3). Repeat blood investigations showed a total white blood count of 8.5 × 10^9^/L and a C-reactive protein of 41.7 mg/L. Patient was then kept nil by mouth and an urgent contrast-enhanced CT of the abdomen and pelvis was done on post-op day 4 to rule out any intraabdominal collections, leaks, or fistulation. However, only post-op changes were noted with no intraabdominal collections or evidence of contrast extravasation to suggest a leak. Once patient was kept nil by mouth, both drains returned to draining hemoserous fluid, suggesting a chyle leak. Drain fluid from both drains were sent for fluid triglycerides, and came back raised at 5.6 mmol/L (101 mg/dL) from the intraabdominal drain, and 6.6 mmol/L (119 mg/dL) from the subcutaneous drain, confirming a chyle leak. Patient was referred to dietitian and started on a very low-fat diet. He was also subsequently started on subcutaneous octreotide 100 μg. Both drains were initially draining about 100-260 mL of chylous fluid per day, which then subsequently reduced to minimal output. Patient was discharged well on post-op day 11 with both drains intact after completing care giver training for the drains.

**Fig. 4:**
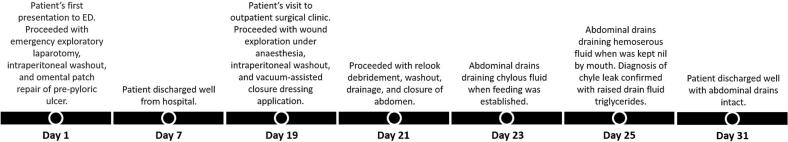
Timeline summary of the sequence of clinical events from first presentation to the emergency department.

Patient was followed up closely in the outpatient surgical clinic. His subcutaneous drain was removed at 1 month post-operatively, while his intraabdominal drain dislodged and fell out about 2 months post-operatively. Patient was kept on regular dressing for his abdominal wound in the outpatient clinic. His last review after almost 5 months post-op noted complete healing of abdominal wound and was subsequently discharged from the outpatient surgical clinic. A timeline depicting the summary of the sequence of clinical events from first presentation to the emergency department until discharge is shown in Fig. 4.

## 3 Discussion

There are various pathologic conditions involving the abdominal lymphatic system that can lead to chylous ascites (Table 1). Post-operative chyle leak usually occurs from the intraoperative disruption of the thoracic duct, cisterna chyli, or major tributaries of the lymphatic system, and hence can also occur following abdominal and retroperitoneal surgeries [9]. These however, are usually more major surgeries that involve or are closely located to the major lymphatic structures. Lymphatic vessels near the stomach follow the arteries into the left and right gastric nodes, the left and right gastroepiploic nodes, and the short gastric nodes. Chyle leakage plausibly could occur with damage to any of these lymphatic vessels or nodes. No reported cases could be found for a chyle leak after an omental patch repair for a perforated gastric ulcer. Russel et al. defines chyle leak as the discharge of milky, lipid-rich (>110 mg/dL) fluid from a surgical wound or drain, which is >100 mL per day in quantity, with the fluid being non-infective and amylase free [9]. Chyle leak can disrupt the complex biochemical milieu that makes up wound healing and lead to delayed wound healing, infection, breakdown, fistula formation, and general sepsis [4].

**Table 1:** Common causes of chylous ascites.

Congenital [3,[13], [14], [15]]		
Congenital idiopathic		
Intestinal lymphangiectasia		
Primary lymphatic hypoplasia		
Chyle cysts		
Lymphangiomatosis		

Acquired [1,3,14,15]		
Neoplastic	Malignant	Lymphoma
		Kaposi's sarcoma
		No lymphomatous
	Benign	
Postoperative	Resection of the abdominal aorta	
	Retroperitoneal lymphadenectomy	
	Pancreaticoduodenectomy	
	Vagotomy	
	Radical nephrectomy	
	Warren shunt	
	Nissen fundoplication	
	Placement of peritoneal dialysis catheter	
Inflammatory	Radiation therapy	
	Tuberculosis	
	Pancreatitis	
	Filariasis/ascariasis	
	Peritoneal dialysis	
	Sarcoidosis	
Traumatic	Blunt	
	Shear force to the root of mesentery	
	Penetrating	
Obstructive	Adhesions	
	Volvulus	
	Intussusception	
	Aortic aneurysm	
Haemodynamic	Cirrhosis	
	Jugular, innominate, left subclavian, or portal vein thrombosis	
	Nephrotic syndrome	

Chyle leakage is not one of the known postoperative complications following an omental patch repair [15]. As chyle leak after omental patch repair has not been reported before, we sought to ascertain the causes. The first postulation was a chyle leak from his prior trauma which he was asymptomatic from [7]. This patient did report being involved in a road traffic accident 2 days prior to presentation to the emergency department. However, patient reported no abdominal injuries. Intraoperatively as well, no chyle was noted and the perforated pre-pyloric ulcer was benign looking suggesting a more non traumatic cause of ulcer perforation. The ulcer edge was biopsied intraoperatively, and histology reported as just benign patchy chronic inflammation. At this juncture, abdominal trauma still remains as one of the plausible causes for the presentation of the chyle leak.

The other postulation was an iatrogenic cause from injury to the lymphatic system intraoperatively. This case presented with a small perforated pre-pyloric ulcer that was anteriorly located. A classical laparotomy, washout and omental patch repair was performed by experienced surgeons. There were no complications that were encountered during the surgery, and hence it seemed less likely that the chyle leak was due to an iatrogenic intraoperative injury to the lymphatic system. It is still, however, a plausible cause for the leak. Possibility of surgical cause in our case is from the injury to these lymph vessels or nodes which could have occurred during the omental manipulation.

**Fig. 5:**
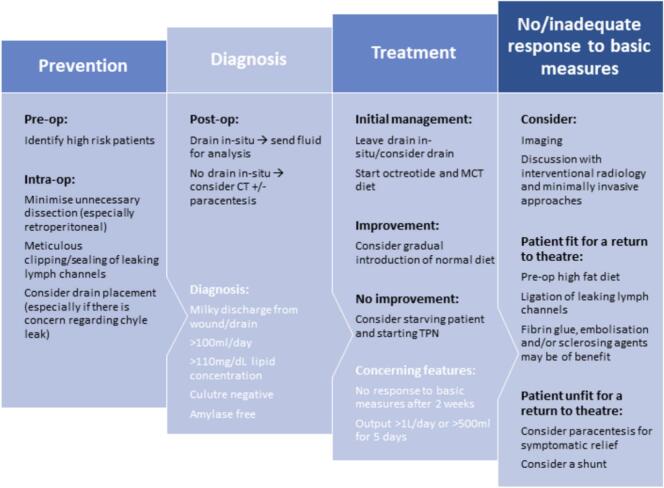
Proposed algorithm for the prevention, diagnosis, and initial management of chyle leak following abdominal surgery [9].

Russell et al. has proposed an algorithm for prevention, diagnosis, and initial management of chyle leak following abdominal surgery (Fig. 5) [9]. The initial management includes leaving any abdominal drains in-situ, starting octreotide, and medium-chain triglycerides diet. Gradual introduction of normal diet is then considered with improvement. If there is no improvement with the initial management, then will need to consider keeping the patient nil by mouth and starting TPN. No, or inadequate response to the basic conservative measures will then prompt the need for further imaging, minimally invasive approaches, or definitive surgery.

This case was successfully treated conservatively. Weniger et al. describes the conservative management of chyle leak is based on the theory that spontaneous closure of the chyle leak will occur with decreasing chyle flow output, with the aim of nutrition therapy to assist with the reduction of chyle output, replacement of fluids and electrolytes, and maintain the nutrition status [16]. A high volume chyle leakage can be defined as an output of more than 1.5 L per day [8]. This patient's output never exceeded 260 mL per day, and is therefore a low volume chyle leak. Literature found that a more conservative approach to management of a chyle leak can be very successful with a success rate ranging from 58 to 100 %. Among this approach includes suction drains, and a low-fat diet that is directly absorbed into the portal circulation which in leads to a reduced lymphatic flow of chyle [4,7,8]. Russel et al. also proposes administration of a somatostatin (growth hormone inhibiting hormone) such as octreotide. Whilst the exact mechanism of action is still unknown, it is thought to reduce lymph production via decreasing the absorption of triglycerides, inhibiting splanchnic circulation, and decreasing gastrointestinal motility. It has also shown to result in earlier resolution of chyle leak. These were taken into consideration when managing this patient, and hence a prompt dietitian review for a strict very low-fat diet, and starting on subcutaneous octreotide was done as soon as the diagnosis was confirmed. Patient was also on continuous drain monitoring whilst inpatient before discharging to follow up of the abdominal drains in the outpatient setting.

Invasive therapies are only indicated if initial conservative measures have failed [9,12]. This is if the chyle leakage is persistent despite more than 2 weeks of conservative therapy, a high output chyle leakage, or if metabolic or immunologic complications are faced [9]. Surgery may be proceeded with either a laparoscopic/laparotomy approach to identify and ligate leaking lymphatic channels, or via a peritoneovenous shunt [9].

Chyle leakage complication after abdominal surgery is rare, but has been reported after multiple types of abdominal surgery, especially after pancreatic and gynaecological surgery [10]. The risk of leakage increases with radical surgery, extensive lymph node dissection, dissection around the paraaortic region, and retroperitoneal dissections. Makal et al. reported a case of a lymphatic leakage after laparoscopic sleeve gastrectomy [2]. This was postulated to be from a possible lymphatic duct injury near the left crus that is always dissected during laparoscopic sleeve gastrectomy, or a lymph node injury. Shen et al. [11] reported a case of chyle leakage after laparoscopic cholecystectomy, however in a patient with duplicated cystic ducts. Although extremely rare, they postulated that there may be a potential relationship between the anatomic variation of the extrahepatic duct and the anatomic variation of the lymphatic system for their case. A classical laparotomy, washout and omental patch repair has never been reported to produce a chyle leak complication compared to other non-oncological abdominal surgeries, such as laparoscopic sleeve gastrectomies or laparoscopic cholecystectomies. This is due to the stomach's anatomical position in relation to the major lymphatic system. A chyle-leak can be prevented by identifying high risk patients pre-operatively, intraoperatively minimising unnecessary dissections (especially retroperitoneal), and early consideration of drain placement if there is a possible concern regarding chyle leak [9]. If any chyle-like fluid is noted intraoperatively there should be a high index of suspicion for a possible chyle leak. This was not noted during the first surgery in our case, and was only noted on the representation to the outpatient surgical clinic, which then manifested as a wound dehiscence from the persistent chyle leakage. Post-operatively, a high suspicion of a chyle leak should be considered if a milky chylous-like fluid is seen in the intraabdominal drains.

## 4 Conclusion

This is the first reported chyle leak after a perforated gastric ulcer repair in the available literature. Causes for the chyle leak from this case include traumatic chyle leak, and iatrogenic injury. In such a case, a low-volume chyle leak can be successfully treated conservatively.

## Author contribution

1. Muhammed Isa Bin Jusoff Albar; wrote the case report, came up with the literature review, and completed the discussion portion of the case report.
2. Han Boon Oh; supervised the writing of the case report and assisted with the discussion portion of the case.

## Consent

Written informed consent was obtained from the patient for publication and any accompanying images. A copy of the written consent is available for review by the Editor-in-Chief of this journal on request.

## Ethical approval

Approval is exempted in my institution (Ng Teng Fong General Hospital, Singapore) as the submitted manuscript is a single case report.

## Guarantor

1. Muhammed Isa Bin Jusoff Albar
2. Han Boon Oh

## Research registration number

Case report submitted is not a ‘First in Man’ study.

## Funding

None to be declared.

## Conflict of interest statement

None to be declared.
